# Molecular and Integrative Physiological Effects of Isoflurane Anesthesia: The Paradigm of Cardiovascular Studies in Rodents using Magnetic Resonance Imaging

**DOI:** 10.3389/fcvm.2016.00023

**Published:** 2016-07-29

**Authors:** Christakis Constantinides, Kathy Murphy

**Affiliations:** ^1^Chi Biomedical Ltd., Nicosia, Cyprus; ^2^Division of Cardiovascular Medicine, University of Oxford, Oxford, UK; ^3^Division of Biomedical Sciences, University of Oxford, Oxford, UK

**Keywords:** anesthesia, isoflurane, nitrous oxide, rodents, cardiac function, inotropy, lusitropy, magnetic resonance imaging

## Abstract

To-this-date, the exact molecular, cellular, and integrative physiological mechanisms of anesthesia remain largely unknown. Published evidence indicates that anesthetic effects are multifocal and occur in a time-dependent and coordinated manner, mediated *via* central, local, and peripheral pathways. Their effects can be modulated by a range of variables, and their elicited end-effect on the integrative physiological response is highly variable. This review summarizes the major cellular and molecular sites of anesthetic action with a focus on the paradigm of isoflurane (ISO) – the most commonly used anesthetic nowadays – and its use in prolonged *in vivo* rodent studies using imaging modalities, such as magnetic resonance imaging (MRI). It also presents established evidence for normal ranges of global and regional physiological cardiac function under ISO, proposes optimal, practical methodologies relevant to the use of anesthetic protocols for MRI and outlines the beneficial effects of nitrous oxide supplementation.

## Introduction

Much has been written on volatile (inhalational) anesthetics and their potential mechanisms of action. Interestingly, to-this-date, and despite great advances in the field, the exact molecular, cellular, and integrative physiological mechanisms of anesthesia remain largely unknown. With the establishment of rodent models as the preferred models for the study of cardiac disease, transgenic modifications, and for an increasing number of functional genomics studies, the effects of anesthesia on rodents have become paramount for basic science. While the understanding of the mechanisms of anesthetic action may be limited, it is critical for normalization and comparative studies (to the very least) that the integrative physiological effects are appreciated and considered in the design and execution of *in vivo* disease studies. Unavoidably, the role of *in vivo* imaging and magnetic resonance imaging (MRI) in biomedical research has been increasing, and it is envisaged to become integral for future multicenter, consortia, and other targeted image-based phenotyping studies.

It is the purpose of this review to (a) summarize the major sites of anesthetic action for *in vivo* studies at the organ and cellular levels, with a focus on isoflurane (ISO), the most commonly used volatile anesthetic (1), (b) present established evidence for the normal ranges of global and regional physiological cardiac function under ISO, (c) propose optimal, practical methodologies relevant to the use of anesthetic protocols for MRI, and (d) outline the beneficial effects of N_2_O supplementation.

We thus present a brief historical overview and a synopsis of the existing knowledge of organ, cellular, and molecular mechanisms of anesthetic action, complemented by experimental evidence on global/regional cardiac function from recent work, in justification of the need to establish and use optimal protocols of study.

## Historical Overview and Current Knowledge on Mechanisms of Anesthetic Action

### The Evolution of Anesthetics – Initial and Current Hypotheses of Mechanisms of Action

The *in vivo* importance of anesthetics (beyond their narcotic, analgesic, and sedative actions) for the study of cardiac function is reflected upon their effects on regional cellular/organ function, and ultimately, upon the elicited integrative physiological response. Despite the reported use of opioids and other naturally occurring analgesics in ancient times (2), the Greek term anesthesia was introduced later on. This composite term indicates the lack of sensation/consciousness (Greek: ανɛυ-αίσθηση) and dates back to the era of the ancient Greek surgeon Dioscorides (3).

In the modern era, nitrous oxide (N_2_O) was the first synthesized anesthetic in the 1770s by J. Priestley (2, 4). It was subsequently used and dubbed by Sir Davy in 1799 as the “laughing gas” (4). It was unsuccessfully used to induce general anesthesia (primarily due to its low potency as a stand-alone anesthetic) by the American dentist Horace Wells in 1845 (2, 4). While multiple other attempts on the use of anesthetics were subsequently reported, most noteworthy was the successful induction of a reversible, insensible state, by Sir Oliver Wendell Holmes in 1846 (3), and W. Morton’s first successful public demonstration of ether-induced anesthesia (5). Inhalational anesthetics were developed and introduced later on. ISO became clinically available in 1972, with a demonstrated lower solubility in blood, than e.g., ether and halothane, and with fast lung elimination rates, therefore leading to more favorable pharmacokinetics for anesthetic induction and recovery.

Numerous quests had been documented early on to identify a single, common biological mechanism of potential targeted action, commonly referred and classified as the “unitary hypothesis/theory” (2, 5). Additionally, independent work by Meyer and Overton led to the hypothesis of existence of a strong correlation between the potency of an anesthetic agent and its lipid solubility (that is, its ability to cause perturbations in the lipid bilayer of cellular and subcellular structures), commonly known as the “Meyer–Overton” rule (6, 7). These two concepts dominated the thinking on the mechanisms of anesthetic action for a considerable time. However, numerous discrepancies based on experimental observations have led the scientific community to largely abandon both concepts. For example, lipid bilayer perturbations have also been documented (2, 5, 8) experimentally *in vitro* by temperature increases of less than 1°C (8). Furthermore, use of anesthetic stereoisomers have elicited differential effects when used *in vitro* and *in vivo*, indicating that the primary site of action is most likely not the lipid bilayer (8). Correspondingly, there exist halogenated alkane compounds (known as “non-imobilizers”) that do not obey the Meyer–Overton rule. As stated by Campagna et al. (5), collectively, the reasoning for such discrepancies may be attributed to anesthetic size, rigidity, polarity variations, and the localization of anesthetics within the bilayer. Remarkably, even Linus Pauling’s physicochemical clathrate formation theory (9) failed to account for the diverse, multifocal, and spatially heterogeneous anesthetic localization and actions. The theory of lipid solubility of anesthetics has thus largely been abandoned, although the hypothesis for gaseous lipid bilayer permeation, diffusion, and bilayer disruption (lipid bilayer–protein interaction) is still under investigation (2, 5).

Scientific focus was thus redirected to the possible interactions of lipids and structural proteins, but a comprehensive, integrative model hypothesis is still lacking (5, 8).

### Anesthetic Induction and Maintenance – Central, Peripheral Pathways, and Targeted Organ Sites

**Figure 1:**
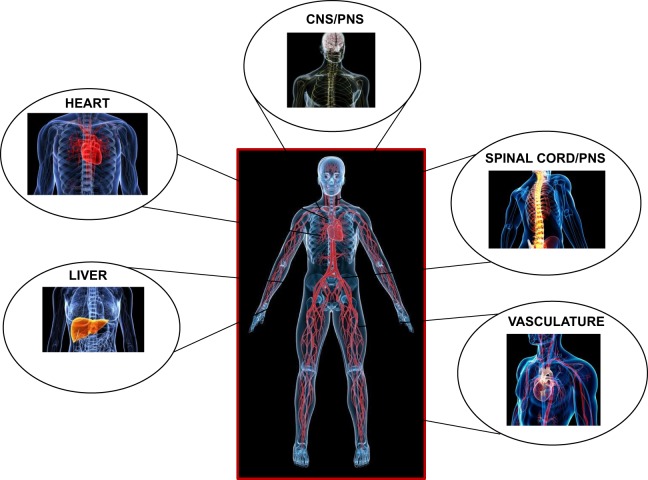
**Major loci of targeted anesthetic action centrally and peripherally**.

Central to their function is their ability to induce a reversible state of unconsciousness. Inhalational anesthetics are believed to be fast to equilibrate from the onset of induction most likely due to the fast exchange at the lung-alveolar/blood capillary interface and the fast diffusional transit to the vascular and interstitial spaces (10). They exert their effects centrally, on the central (supraspinal centers and the cortex) and the peripheral nervous systems (spinal cord and neuronal processes), and on the heart, liver, and vasculature, in a temporospatial pattern that is likely different during induction and recovery (11) (Figure 1).

While the validity of the cognitive binding/unbinding theories for the disruption of frontoparietal cortical communication (12, 13) or imbalances of the excitatory/inhibitory neuronal transmission are still under dispute (14, 15), it is currently believed that the foundation of unconsciousness is exemplified by the integrative disturbance of neuronal pathways, involving the reticular formation in the brain stem and supratentorial signaling paths, through the mediation of junctional thalamic and cortical regions (2, 16). It is also unknown on whether descending cortical or ascending spinal signals exert differential effects on the hypnotic action of anesthetics, and if these are dose dependent (5). However, what is well established is the spatial distribution of inhalational anesthetic accumulation based on tomographic PET studies, indicating significant dose-dependent accumulation of ISO in the brain, heart, and liver regions, in support of coronary and cerebral blood flow (CBF) increases, and an altered metabolism (17) (Figure 1).

### Molecular Mechanisms of Anesthetic Action *via* Cellular and Receptor-Mediated Signaling Pathways

The molecular basis of anesthetic action involves membrane proteins (2, 5, 18–21, 110). Primarily and foremost, molecular mechanisms are synaptic, affecting the cysteine-loop superfamily of neurotransmitter receptors and mediating either increases of inhibitory postsynaptic excitability or decreases of presynaptic (excitatory) neurotransmitter release. Key molecular targets are involved in the establishment of unconsciousness and the maintenance of hypnotic action, either directly targeting spinal and supraspinal centers or cellular entities in the heart and liver, and in the peripheral vasculature. Given the disparity of receptor types, density, distribution, sensitization, types of binding sites and affinities, low-affinity anesthetic interactions, and differential effects to induction versus recovery, the exact molecular effects of mediated action are still unclear. While possible gene expression effects have not been investigated in detail, most studies have addressed changes in the total conductance of ion channels as determined by changes in their innate conductivity and open–close probabilities.

**Figure 2:**
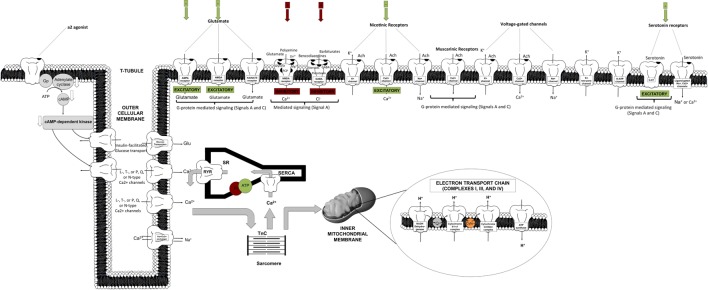
**Schematic representation of targeted cellular loci of anesthetic action**. This cartoon representation combines receptor-mediated processes specific to cortical, spinal cord, cardiomyocyte, liver, and vasculature sites [AMPA, amino-3-hydroxy-5-methyl-4-isoxazol-propionic acid; Cyto, cytochrome c; GABA, gamma-aminobutyric acid; NMDA, *N*-methyl-d-aspartate; PL, phospholamban; Q, quinone; RyR, ryanodine receptor; SR, sarcoplasmic reticulum; SERCA, sarcoplasmic endoplasmic reticulum calcium pump; TnC, troponin C].

The primary inhibitory channels that are likely affected by anesthetics include the chloride channels relevant to the gamma-aminobutyric acid (GABA)_A_ and glycine receptors and two-pore potassium (K_2P_) channels, including voltage-gated (K_v_) and adenosine triphosphate (ATP) potassium (K_ATP_) channels (2, 5, 8, 22, 23). Figure 2 depicts in a comprehensive and integrative manner the receptor types mediated in anesthetic inhibitory/excitatory action (spanning cellular sites in the brain, heart, liver, and other targeted regions). Evidence also exists for the modulation of the chlorine channel conductance by volatile anesthetics, possibly *via* indirect action, and most likely dependent on Ca^2+^ changes (22).

On the other hand, excitatory inhibition targets ligand-gated nicotinic, muscarinic, amino-3-hydroxy-5-methyl-4-isoxazol-propionic acid (AMPA), kainate, *N*-methyl-d-aspartate (NMDA), and serotonin (5-HT2 and 5-HT3) receptors (2, 5, 8). Other targets include voltage-gated channels, spanning Ca^2+^ channels [L, T, or N and P/Q types, or ryanodine–inositol triphosphate (RyR–IP_3_) receptors], K^+^ channels (inward rectifiers), or fast activation/inactivation channels (including Na^+^ channels). These seem to be affected in a diffuse, non-spatially specific manner (negative inotropy and chronotropy). Potassium channels have also been shown to be involved in the effective action of a_2_-agonists (centrally or peripherally).

#### Intracellular Signaling

**Figure 3:**
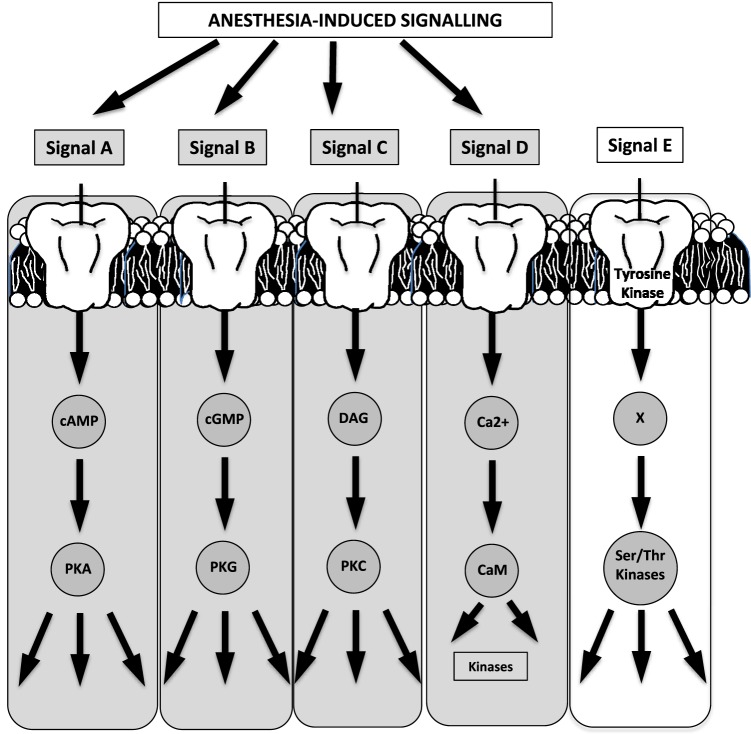
**Four of the five known major signaling pathways are triggered by anesthesia (the schematic has been generated based on a relevant schematic in Guyton’s textbook on human physiology)**. For example, effects may be potentiated (signal A) on GABA_A_ or a2-receptors, while ion–channel binding may potentiate second-messenger effects (signal D) [cAMP, cyclic adenosine monophosphate; cGMP, cyclic guanosine monophosphate; DAG, diacyl glycerol; PKA, protein kinase A; PKG, protein kinase G; PKC, protein kinase C; CaM, calmodulin; Ser, serine; Thr, threonine].

A reasonable extrapolation of prior evidence for targeted anesthetic sites (see prior section) involves pathways and processes downstream of cell surface receptors and ion channels. In spite of the difficulty in identifying specific anesthetic binding sites (as a result of the low-affinity interactions of inhaled anesthetics and the lack of knowledge of atomic structures of ion channels or membrane proteins), existing evidence stems from work on proteins with known structures (e.g., albumin and lusiferase) (24, 110) or has identified signaling binding sites in protein amino acids within neurons (25), protein kinases (26), or in mitochondrial complexes III and V (27) (Figure 2). Figure 3 summarizes the five known, major signaling pathways (28), executed through second messengers, protein phosphorylation, or G-protein mediated pathways.

Highlighted in Figure 3 are the four cascades that are likely triggered or affected by anesthetics (29, 110, 115) with indicative signals identifying GABA receptors for cyclic adenosine monophosphate (cAMP)-mediated phosphorylation, glutamate receptors for G-protein mediated signaling, and a G-protein coupling of a2-adrenergic receptors/agonists.

Anesthetics induce specific potentiation of the activity of protein kinase C (PKC) and non-specific inhibition of protein kinase A (PKA) (30). Although the spatial distribution and subtypes of a2-receptors in rodents are poorly characterized (central versus peripheral, pre- versus postsynaptic), there are at least three receptor subtypes. Effectively, the molecular signaling mechanism is initiated by a2-agonist binding, phosphorylation of G-mediated protein G_p_, phosphorylation of adenylate cyclase, and downregulation of cAMP, thereby leading to downregulation of cAMP-dependent kinase and ion–channel conductance alteration, ultimately causing hyperpolarization (Figure 2). The posttranslational phosphorylation of proteins on serine, threonine, or tyrosine groups also seems to be plausible (110).

In summary, the primary molecular mechanism of anesthetic action is not only the alteration of the chlorine’s channel conductance but also the conductance of other channels (Ca^2+^, K^+^) through spatially dependent action in the brain, heart, and other target organs, and the periphery. In addition, there are documented receptor-mediated channel (*via* cAMP or PKC phosphorylation) and G-protein mediated signaling cascade effects.

## Cardiovascular Effects of Isoflurane and Integrative Physiological Effects

Anesthetics have been shown to cause moderate to severe cardio-depression (31–38) with adverse physiological effects manifested through dose-dependent contractile changes on the heart, brain, and the vasculature, also affecting hormonal release (39–41), perfusion, and metabolism (42–45). This section briefly summarizes such effects at the global and regional cardiac functional levels and through integrative physiological action.

### Cerebral, Myocardial Perfusion, and Coronary Blood Flow

Isoflurane is a potent cerebral (46, 47), coronary (113), and peripheral vasculature vasodilator (43, 44). Furthermore, it also likely abolishes (in a dose-dependent manner) sympathetic tone, although published results from Ebert and Stowe (49) suggest that this effect may be spatially heterogeneous, given evidence for preservation of sympathetic vasomotor activity.

Spatially heterogeneous CBFs were documented non-invasively in rodents using arterial spin labeling with MRI (46, 47, 111). In these studies, preferential vasodilation in thalamic versus other cortical regions (47) was evidenced, in association with documented mean arterial blood pressure (MAP) reductions.

While CBF is tightly regulated by local (myogenic, endothelial, metabolic) and systemic (autonomic, humoral) factors (47), and although its changes may be mediated by an interplay of numerous factors (43), nitric oxide (NO) release is likely involved in the case of volatile anesthetics (46, 47, 50). In justification of this mechanism was evidence for increased cerebral expression levels of NO synthetases post-ISO administration (51). However, of interest may be the dose-dependent MAP decreases, the differential NO expression levels in the heart and peripheral vasculature, and autoregulatory or feedback mechanisms for MAP baroreceptor set-point depression and stabilization.

Prior PET studies have documented spatial perfusion changes in a dose-dependent, serially temporal, uptake manner of ISO in the brain, heart (17), gland tissue, kidney, intestine, and liver (52), indicative of coronary, cerebral, and other organ blood flow increases and altered metabolic profiles.

In fact, significant increases of myocardial blood flow (MBF) have been observed under ISO in numerous invasive (48) and MRI studies (53) in swine (54), open-chest dogs (55, 56), and in isolated (57, 58), and intact rat hearts (53). The coronary vasodilatory effects of ISO have been shown to be vascular smooth muscle and endothelium-dependent (59), and mediated by K_ATP_ channels (60, 61). Although the exact mechanism of action is unknown, Crystal et al. (48) have suggested the possibility of (a) direct involvement of halogenated anesthetics (sevoflurane, desflurane) with K_ATP_ channels, (b) ATP decreases in vascular smooth muscle cells causing K_ATP_ channel opening, (c) a prostacyclin-triggered G-protein mediated pathway and K_ATP_ channel opening, or (d) adenosine receptor-G-protein, or (e) PKC phosphorylation and channel activity modulation.

Overall, it is argued that the net effect of inspired anesthetics on CBF may be relevant to the net effect of their vasodilatory action versus time-dependent vascular adaptation and metabolic mechanisms secondary to a reduced cardiac workload/state (48).

### Metabolism

Significant reductions in heart rate and lesser reductions in MAP from the conscious state are manifested in rodents as a result of the use of inhalational anesthetics. As stated above, the potent vasodilatory role of some of these anesthetics (e.g., ISO) lead to increased blood flows in several vascular beds (17, 52, 62, 114). Although changes in the myocardial oxygen extraction (EO_2_) reflect the compound effect of metabolic and vascular changes postanesthesia administration, its decreased value post-induction (48) hints to possible alterations of metabolic status. Correspondingly, the major determinants of myocardial oxygen consumption (MVO_2_) are myocardial contractility, wall tension, and heart rate. MVO_2_ is thus often correlated to the product of HR and MAP and regional contractility (48), and it is expected to be decreased, but potentially maintained constant over prolonged anesthesia exposure (43, 44). This decrease (in association to oxygen extraction), directly reflects contractile changes (to be discussed below) or metabolic changes, manifested by reductions in the efficiency of ATP production by the mitochondria, or both. As reported earlier by Constantinides et al. (43), numerous prior rodent studies have shown that inhalational anesthetics decrease glucose metabolic rates as a result of the inhibition of ATP synthesis (63), thereby leading to the uncoupling of oxidative phosphorylation (23, 27). Impaired glucose tolerance, increased glucose, insulin, and glucagon levels have also been reported (as a result of altered hepatic metabolism and enzyme activity) (43, 64, 112). Sympathetic tone and immediate hormonal release postanesthesia induction (65) lead to a rapid hyperglycemic effect, results that have been observed independently by Constantinides et al. in C57BL/6 mice (43). It is unknown on whether such effects are also mediated *via* direct anesthetic action on the membrane’s glucose/insulin transporters (Figure 2).

### Global and Regional Cardiac Function

#### Effects of Isoflurane on Global Cardiac Function

**Figure 4:**
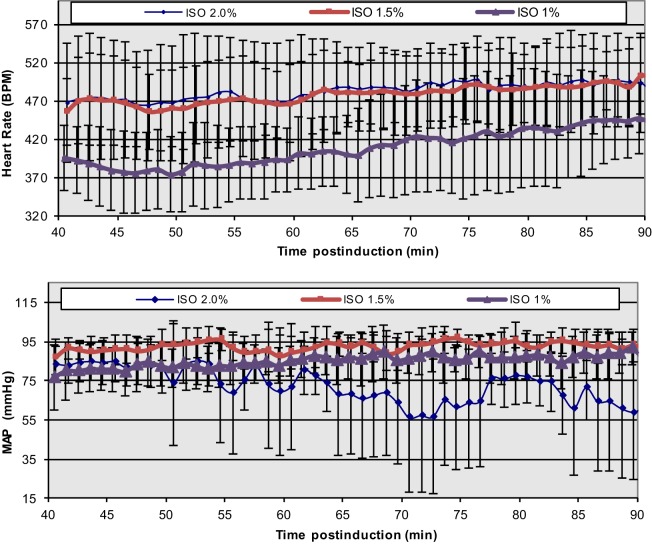
**Temporal dependence of ISO effects at different concentrations on HR and MAP in C57BL/6 mice**. The onset of displayed values has been chosen to match the end of the surgical insertion of the recording sensors and the thermal stabilization of the mice [Reproduced and adapted from Constantinides et al. (43). By permission from Oxford University Press on behalf of the Institute for Laboratory Animal Research. This material is published under a Standard License. Forward reuse is prohibited. For permission, please contact journals.permissions@oup.com].

The extreme discrepancy between basal cardiovascular function in the conscious and anesthetized states in rodents requires care in the design and execution of physiological, transgenetic, and pathological cardiac studies, especially in cases of non-invasive MRI, where prolonged anesthesia is required. In conjunction with anesthesia-induced flow reductions, metabolic downregulation, the extreme bradycardic state, and a possible altered cardiac functional *modus operandi*, the assessment of optimal induction and anesthetic administration and maintenance over prolonged time periods (e.g., ≥90 min) that match most conventional MRI protocols becomes critical. In our prior study (43, 44), we have accomplished a highly reproducible and stable mouse maintenance protocol (beyond 40 min) under ISO at optimal levels of approximately 1.5% [mean HR = 478 ± 11.4 and coefficient of variability (CV) = 11 ± 1%; mean MAP = 92 ± 2 mmHg, CV = 8 ± 2%], following the end of the surgical procedure for sensor placements and the reestablishment of thermal stabilization (Figure 4).

While lower concentrations of administered ISO (1%) may be physiologically preferred, more erratic and highly variable states have been observed. Certainly, higher % doses must be avoided in order to avoid severe cardio-depression.

Overall, the observed bradycardic state may be associated with direct anesthetic effects on electrical sinoatrial/atrioventricular nodal activity and action potential propagation, or possibly reflective of the metabolic downregulation and the reduced sympathetic tone (66, 67). The smaller reductions in MAP (compared to the conscious state), which is directly dependent on the product of cardiac output (CO = HR × SV) and total peripheral resistance (TPR), indicate a well-controlled, systemic, and integrative regulation of the blood pressure, and overall increases in TPR. The latter are likely manifested by either ISO-related vasomotor effects (given the vasodilation observed in different vascular beds) and/or blood flow redistribution (43). Shunting of blood from one vascular bed to another is extremely fast in the mouse and can occur within a period of seconds.

#### Regional Cardiac Inotropic and Lusitropic Effects of Isoflurane

Regional cardiac contractile status (inotropy, lusitropy) is associated with HR, cross-bridge cycling, sarcomeric force generation, and calcium dynamics. Calcium channel entry, sarcoplasmic reticulum (SR) storage, calcium-induce and calcium-release *via* RyR receptors, troponin C (TnC) binding, cross-bridge formation and cycling, and SR pump (SERCA) sequestration are all deterministic of calcium homeostasis (Figure 2).

Isoflurane-induced cardio-depression is manifested by chronotropic and inotropic decreases. Modulation of the autonomic tone (sympathetic system) mostly accounts for chronotropic effects. Correspondingly, decreases of the inward calcium transient and SR accumulation/release lead to inotropic changes. The potential role of the Frank–Starling law and the force-frequency reserve (68–71) has been shown to be less important in mice due to their minimal effects. Prior studies in canine and pigs with ISO (31, 36) attribute dose-dependent, anesthetic-induced contractility decreases to diminished contractile protein sensitivity (TnC) to calcium binding, altered myofilament responses, and to the SR release and reuptake processes (Figure 2).

However, a recent study by Ding et al. (72) in intact and skinned right ventricular trabecular muscles in the rat has provided evidence in support of the ISO-induced cardiac force depression as a direct result of the decreased myofilament responsiveness to Ca^2+^. The authors also convincingly argue on the possible regulatory site of ISO action, attributed primarily on the tropomyosin–actin complexation, as mediated by the exposure of actin–myosin binding sites during excitation–contraction or by modifying soluble cytoplasmic factors that may modulate the myofilament’s behavior. The same study shows that myofilament Ca^2+^ sensitization by the nitroxyl (HNO) donor 1-nitrosocyclohexyl acetate (NCA) reverses ISO-induced effects and effectively restores force development without any concomitant increases in the intracellular calcium (72). Complementary to this study, Fukuto et al. (73) summarize HNO effects in different species, attributing the augmentation of Ca^2+^ release and reuptake from the SR in both murine cardiac and skeletal muscles to ryanodine receptors and SR Ca^2+^ ATPase activation (74). It is also argued that calcium reuptake in the SR is stimulated by HNO (74). Thus, taken together, it is currently unknown on whether the mechanism for nitroxyl’s action *in vivo* is *via* a direct effect on the contractile proteins and/or modulated by calcium SR cycling, or whether species-dependent effects are present.

Regional, *in vivo* myocardial contractile performance can be characterized based on numerous quantitative, load-independent, and dependent hemodynamic indices, obtained from pressure–volume catheterization studies. Pacher et al. (75) have published catheterization protocols for various anesthetics (including ISO) and proper hemodynamic ranges of contractile function (Table 1). Although these ranges reflect open and closed chest preparations and short-term anesthetic exposure, Constantinides et al. (43, 44) have extended the study of global functional indices (Figure 4) (44) under optimal and prolonged anesthetic conditions (up to 90 min post-induction) to the study of regional functional indices (Table 1). These results are in close agreement or within the ranges reported by Pacher et al. (75) and indicate constancy of contractile and relaxation performance at the regional fiber level, most appropriate for prolonged MRI cardiac imaging studies.

**Table 1:** **Summary of reported ranges of normal, regional, physiological cardiac indices from invasive catheterization studies using different anesthetics**.

Cardiac mechanical index	Reported catheterization results^a^	Reference
Heart rate (HR) (bpm)	470–620	Pacher et al. (75)
	581 ± 23	Shioura et al. (76)
	455 ± 59	Joho et al. (77)
	482 ± 43	Reyes et al. (78)
	505 ± 19	Yang et al. (79)
	634 ± 14	Georgakopoulos et al. (80)
	475.5 ± 17.9	^b^Constantinides et al. (44)
End systolic pressure (ESP) (mmHg)	92–118	Pacher et al. (75)
	93 ± 2	Shioura et al. (76)
	84 ± 10	Reyes et al. (78)
	93.5 ± 4.3	Yang et al. (79)
	112.1 ± 4.3	Georgakopoulos et al. (80)
	102.8 ± 1.5	Constantinides et al. (44)
End diastolic pressure (EDP) (mmHg)	1–6	Pacher et al. (75)
	7.2 ± 1.0	Shioura et al. (76)
	10.2 ± 3.2	Reyes et al. (78)
	5.3 ± 0.8	Georgakopoulos et al. (80)
	15.5 ± 0.6	Constantinides et al. (44)
End systolic volume (ESV) (μl)	7–21	Pacher et al. (75)
	20 ± 2	Shioura et al. (76)
	18 ± 7	Reyes et al. (78)
	11.8 ± 1.1	Constantinides et al. (44)
End diastolic volume (EDV) (μl)	25–53	Pacher et al. (75)
	33 ± 10	Reyes et al. (78)
	15.4 ± 1.1	Yang et al. (79)
	22.3 ± 1.2	Constantinides et al. (44)
Stroke volume (SV) (μl)	17–30	Pacher et al. (75)
	18.2 ± 1.7	Shioura et al. (76)
	18 ± 7	Reyes et al. (78)
	8.3 ± 0.7	Yang et al. (79)
	14.3 ± 0.3	Constantinides et al. (44)
Ejection fraction (EF) (%)	55–72	Pacher et al. (75)
	47.5 ± 2.9	Shioura et al. (76)
	53.4 ± 9.9	Reyes et al. (78)
	54.7 ± 3.3	Yang et al. (79)
	58 ± 14	Georgakopoulos et al. (80)
	59.1 ± 2.0	Constantinides et al. (44)
Cardiac output (CO) (ml/min)	8–16	Pacher et al. (75)
	10.3 ± 7.2	Shioura et al. (76)
	8.9 ± 3.3	Reyes et al. (78)
	6.8 ± 0.3	Constantinides et al. (44)
dP/dt_max_ (mmHg/s)	8200–14,200	Pacher et al. (75)
	9861 ± 624	Shioura et al. (76)
	8738 ± 1659	Reyes et al. (78)
	15,967 ± 809	Yang et al. (79)
	11,777 ± 732	Georgakopoulos et al. (80)
	7912.1 ± 322.6	Constantinides et al. (44)
dP/dt_max_/EDV (mmHg/s/μl)	180–470	Pacher et al. (75)
	343.6 ± 30.3	Constantinides et al. (44)
dP/dt_min_ (mmHg/s)	6700–10,500	Pacher et al. (75)
	−8633±353	Shioura et al. (76)
	−6857±990	Reyes et al. (78)
	−17,297±1367	Yang et al. (79)
	−10,369±909	Georgakopoulos et al. (80)
	−8162.1±355.4	Constantinides et al. (44)
Stroke work (SW) (mmHg/μl)	1500–2600	Pacher et al. (75)
	1349 ± 78	Shioura et al. (76)
	598 ± 68	Yang et al. (79)
	993.0 ± 29.2	Constantinides et al. (44)
Preload adjusted maximum power (PAMP) (mW/ml^2^)	237.6 ± 29.7	Constantinides et al. (44)
Arterial elastance (E_a_) (mmHg/μl)	3–7	Pacher et al. (75)
	5.3 ± 0.6	Shioura et al. (76)
	5.4 ± 2.6	Reyes et al. (78)
	9.4 ± 1.2	Yang et al. (79)
	7.4 ± 0.2	Constantinides et al. (44)
Weiss relaxation constant (τ_weiss_) (ms)	4.4–7.6	Pacher et al. (75)
	8.7 ± 0.4	Constantinides et al. (44)
Glantz relaxation constant (τ_glantz_) (ms)	7–12	Pacher et al. (75)
	8.2 ± 0.7	Shioura et al. (76)
	3.7 ± 0.2	Yang et al. (79)
	6.2 ± 0.5	Georgakopoulos et al. (80)
	11.5 ± 0.6	Constantinides et al. (44)

*The recent studies by Constantinides et al. and Pacher et al. target ISO-induced mouse studies [from Constantinides et al. (44); reproduced from Annals of Biomedical Engineering with permission]*.

*^a^Reported values are either min–max ranges or mean ± SD*.

*^b^Reported values are mean ± SD based on the use of ISO at 1.5% over prolonged periods of anesthesia (40–90 min)*.

## The Beneficial Use of Nitrous Oxide as a Balancing Agent

### Global Organ, Peripheral Stabilization, and Molecular Effects

Despite its long-lived existence (since the early days of anesthetic work) and extensive clinical applicability, the attention on the use N_2_O in basic science has languished and has been disproportionately minimal. Its clinical use has been associated with the effect of a “second anesthetic.” In effect, the use of N_2_O has been shown to have analgesic properties in humans and other species, so it is possible to reduce the administered dose of the primary (volatile) agent and thus minimize its side effects. However, this has been disputed in the case of mice (81). Prior work has indicated that it may exert both cardiac and vascular effects. Price (82) has shown that it enhances sympathetic activity, counteracting the abolition of the sympathetic tone due to ISO, results that are in agreement with Becker and Rosenberg (83), indicating a mild myocardial contractility depression offset by activation of the sympathetic system. Its synaptic anesthetic, analgesic, and nociceptive CNS effects are still questioned (84). Nevertheless, more prominent have been the reports supporting vasoconstrictive action (both systemic and local) leading to vascular resistance, MAP increases (85–87), and stabilization of MAP through prevention of vascular cyclooxygenase expression (87). While human studies report N_2_O-mediated vasodilation subject to increases in plasma homocysteine and decreases of endothelial function (88), it appears that its beneficial effects on sympathetic tone offset such impairments.

Importantly, and in line with the above, are the aforementioned beneficial effects of HNO reported by Ding et al. (72) in conjunction with reports indicating its rapid dimerization to N_2_O (73). Whether the potential added benefits of HNO *in vivo* are direct, and/or mediated *via* the synthesis of N_2_O and its action, are still unknown.

**Figure 5:**
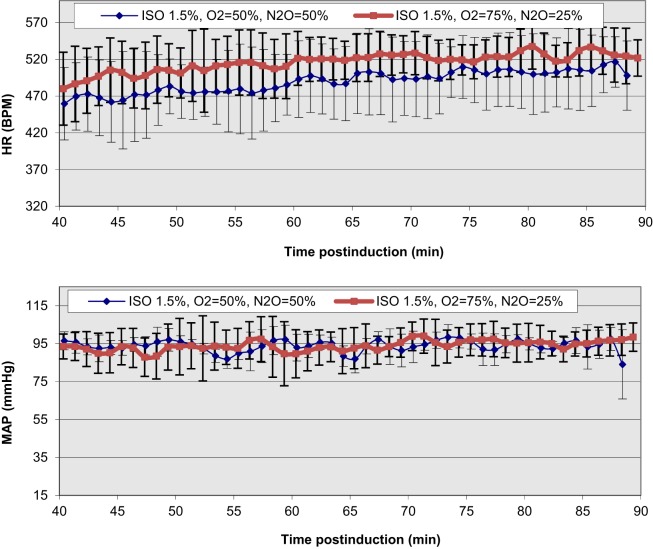
**Functional improvements and temporal stabilization of elicited physiological responses as a result of N_2_O supplementation in the anesthetic ISO mixture in C57BL/6 mice**. The onset of displayed values has been chosen to be after the completion of the surgical insertion of the recording sensors and the thermal stabilization of the mice [Reproduced and adapted from Constantinides et al. (43). By permission from Oxford University Press on behalf of the Institute for Laboratory Animal Research. This material is published under a Standard License. Forward reuse is prohibited. For permission, please contact journals.permissions@oup.com].

Our recent study on C57BL/6 mice (43) has documented prolonged, consistent, and significant benefits from its supplemental use with ISO administration, both in terms of the MAP and HR values, and their beat-to-beat variability (Figure 5). Such findings have been documented in early work at the National Institutes of Health and reproduced in independent catheterization (44) and invasive physiological MLP transgenic studies (89). Given the lack of proven analgesic effects, the effective supplementation of N_2_O concentration can be much lower than normal, typical, clinical levels of approximately 70%. For optimal ISO target doses of approximately 1.5 and 75/25% O_2_/N_2_O supplementation, we have documented 8% increases in mean HR over prolonged periods of study (spanning 90 min post-induction) and 2% MAP increases compared to mean basal activity at 1.5% ISO mixed in 100% O_2_ (43). More importantly, consistent benefits have been documented on the overall variability of mean HR in these time periods, yielding decreases in the CV of 36%. The practical benefits of such outcomes justify an anticipated applicability of these study protocols in non-invasive studies using diagnostic modalities (including MRI, microCT, microPET, and ultrasound).

A direct extension of such findings is also expected to have impact on the study of the overall heart rate variability (HRV), a long-term predictor/biomarker of arrythmogenicity. HRV analyses have been implemented and used in mouse phenotypic screening studies of normal and transgenic mice, as well as the studies of the effects of pharmacologic intervention on the intrinsic heart rhythm and arrythmogenesis (90–92). Standardization and interpretation of HRV results in mice has been difficult due to the disparity of physiological demographics (age), other variables (posture), but primarily due to circardian and physiological variability (93). To this effect, the quantitative measure of the root-mean-square value of the mean squared differences between adjacent normal R–R intervals (RMSSD) has been studied at various fractional oxygen (FiO_2_) delivery and N_2_O supplementation levels (45, 94). Noted was the balancing effect of N_2_O, despite an observed increase in HR variability (compared to the equivalent FiO_2_ study) with time post-induction. Nevertheless, elicited HRV results compare well with prior reported values in FVB mice under ISO (RMSSD = 7.86 ± 0.86) (90), although the exact mechanisms responsible for the noted effects are still unknown.

### Integrative Physiological Effects of Anesthetics

**Figure 6:**
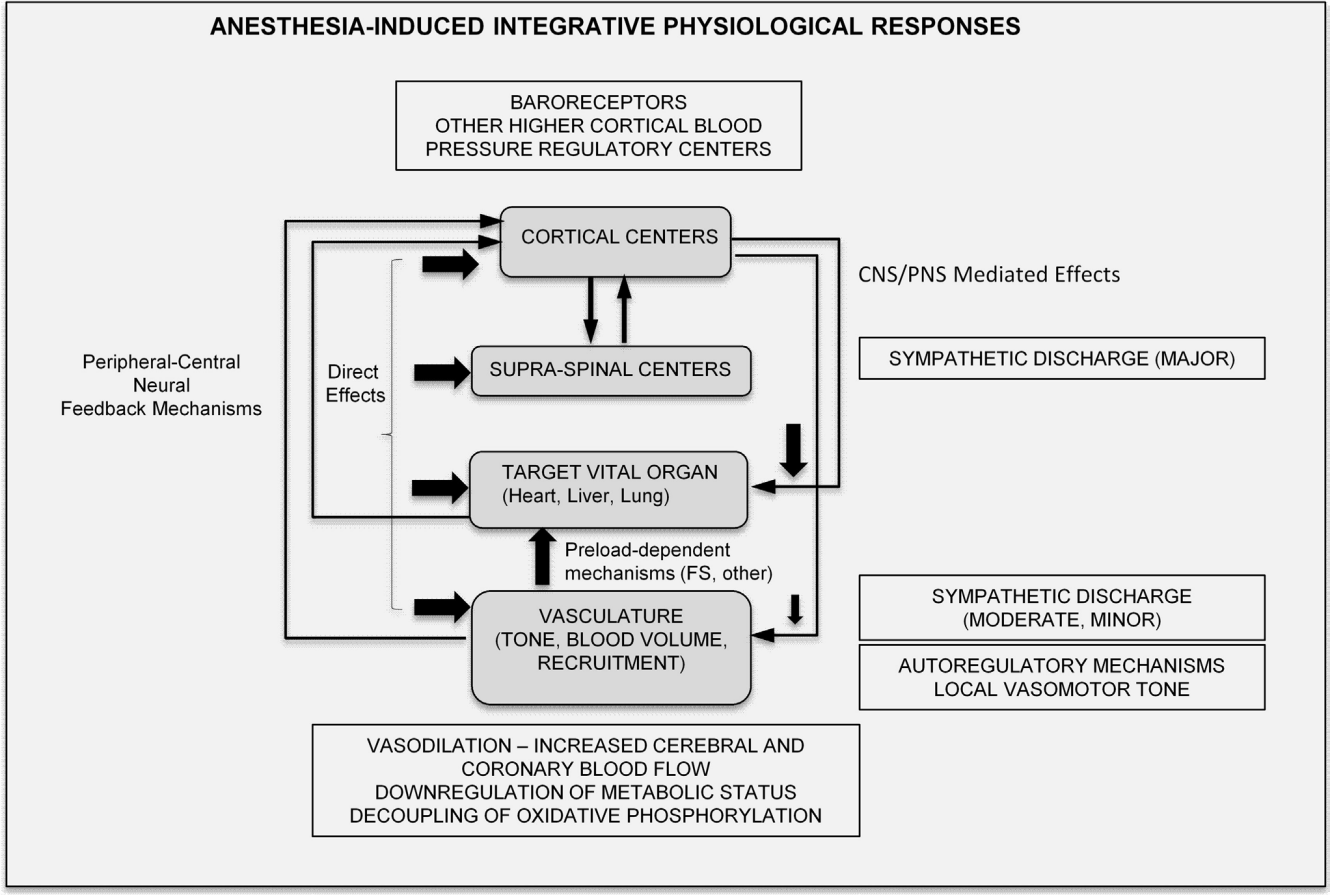
**Proposed integrative physiological responses of anesthesia effects (FS, Frank-Starling; CNS, central nervous system; PNS, peripheral nervous system)**.

In summary, anesthetic effects are multifocal and occur in a time-dependent/coordinated manner, mediated *via* central and local peripheral pathways. Their effects can be modulated by a range of variables (such as strain, age, body temperature, and metabolic status), and their end-effect on the integrative physiological response is highly variable, and thus potentially speculative. Nevertheless, drawing on prior published knowledge, the major, integrative physiological effects of anesthetics can be summarized in the schematic of Figure 6.

## MRI of Cardiac Function Under Isoflurane

### Rodent Phenotyping of Cardiac Function

Cardiovascular MRI of rodents emerged in the 1990s as a natural extension of international efforts conducted to map the human and mouse genomes (completed in 2001 and 2003, respectively), led by the National Institutes of Health. Early studies (95–101) progressed through scalability of equipment and hardware used for humans and adaptability of existing cardiac imaging pulse sequences (conventional gradient echo, spin-echo, and later on steady state at free precession) to the mouse/rat. It was soon realized that dedicated, high-precision sensors, equipment, and high-field systems were required for the non-invasive study of cardiac function, perfusion, and viability in rodents.

Retrospectively, collective efforts in the study of *in vivo* cardiac function targeted global (black and bright-blood CINE) (98, 101–104) and regional contractile responses (strain and velocity imaging) (104–107) in the adult and early embryonic stages, in a scientific attempt to progress high-throughput, image-based phenotypic screening of wild-type, transgenic, and pathological models of cardiac disease. Studies in rodent perfusion and viability effectively developed in parallel with corresponding human studies.

Despite these early advancements in the field and the emergence of multiple international consortia for integrative evaluation and screening, progress has steadily declined. The envisaged future role of image-based phenotyping will entail development of regional, molecular, and intrinsic imaging biomarkers that will be sensitive to early contractile dysfunction, and that will also allow the investigation of spatiotemporal patterns of phosphorylation activity, gene expression, and regional/global contractile response. To this extent, a closer scientific engagement of genome and imaging scientists is envisaged. However, the pace of imaging developments currently lacks compared to genetic technological advancements.

Correspondingly, the conduct of imaging studies in dedicated MRI systems requires adjustments compared to bench-physiological studies, including computer-controlled electrocardiograms and breathing, temperature monitoring systems, and MR-compatible (fiber-optic, electronic, or pneumatic devices, and sensors) and specially designed (with or without negative feedback) thermoregulation systems for efficient bore air-heating. Dedicated mouse cradles (custom-made or commercially available) have also emerged to fit dedicated micro radiofrequency (RF) probes, equipped with dedicated nose cones for delivery of the anesthetic agent, and connected to specially designed ventilators.

Inevitably, current and future imaging studies are, and will be, conducted under anesthesia. Accumulated experience over the last two decades indicates that inhalational anesthetics will continue to be the preferred choice for prolonged image-based phenotyping. Their effects can be potentially detrimental, especially for cases of prolonged administration. The following section is a brief overview of practical considerations and pitfalls relevant to studies under anesthesia.

### Practical Considerations and Anesthetic Protocols

Maintenance of the mouse (or rat) under optimal physiological conditions for 60–120 min of anesthesia administration for MR imaging is a challenging task. Extreme care must thus be exercised for induction, proper anesthetic dose delivery, appropriate acclimation of the animal to its imaging environment, and careful control and monitoring of its temperature and vital signs.

#### Induction

Typical ISO doses range from 3 to 4% over a period of approximately 2–3 min (until the loss of the righting reflex). Excessive and prolonged administration at this dosage level likely leads to an initial oversaturation of receptors with ISO causing excessive physiological depression that may be difficult to reverse subsequently, for instance, if the animal becomes hypothermic.

#### Surgical Preparation

If dedicated sensors are planned to be used, and surgery is to be conducted, it is highly advisable that this takes place as fast as possible. Normothermic conditions must be maintained for the remaining part of the study and for recovery.

#### Anesthetic Maintenance and Recording Sensors

All necessary sensors must be efficiently and properly placed in the minimum amount of time with ISO maintenance at 1–1.5%. If the anesthetic level is set to a higher level, this may induce a cardio-depressed state that is difficult to reverse at later times, even by decreasing the ISO dosage to lower levels (43). This may be related to the anesthetic binding and receptor sensitization, or an initial higher cortical MAP set-point depression that is difficult to reverse. If, however, such a low-dosage level leads to rodent hypersensitivity, agitation, and a light plane of anesthesia, it is recommended that the dose is slightly (but temporarily) increased to approximately 1.75%, before it is reduced to the expected level of approximately 1.5%.

Given the beneficial effects of N_2_O, it is highly recommended that ISO is supplemented with 25% N_2_O, mixed with 75% O_2_. Active pumping of administered N_2_O ensures its release to the environment. The highly beneficial physiological effects from N_2_O use outweigh costs and technical complexity in this case.

Additionally, enforcement of principles of refinement requires use of MR-compatible carbon fiber (adhesive) electrodes, compared to conventional needle ECG. From a technical standpoint, non-metallic electrodes eliminate induced currents and interference during MR gradient pulsations.

#### Anesthesia Administration during the Imaging Study

It is highly recommended that variation of the anesthetic dose during the imaging study and after the initial steady state has been reached is avoided. It is highly risky to perturb the dose level since it often (and easily) leads to cardio-depression. From a physiological standpoint, perturbations likely lead to changes in the anesthesia-receptor sensitization, temporal changes in the anesthetic binding–detachment, perturbations of the steady state, and induce transient changes that ultimately lead to increased physiological variability and inability to stabilize the animal. It is highly preferred that a proper, stable, steady, and deep anesthetic level is reached early on, and maintained at proper conditions, and without a need to modify anesthetic level during the study.

#### Thermoregulation

Given the low bore temperatures and use of open-bore MRI systems, thermoregulation is challenging. Bench studies indicate that the time constant of temperature loss in the mouse is much shorter compared to the time constant for attaining proper physiological temperature. Even with efficient negative feedback systems, it may take several minutes to reach normal temperature levels. In contrast, shunting of blood (hyperemic responses) and temperature loss (due to the large body surface area) is extremely fast and occurs within seconds with important potential implications in beat-to-beat blood volume regulation and thermoregulation.

#### Maintenance of Normal Physiological Status

Although most custom-made and commercial systems are based on HR and respiration indices for ascertaining proper physiological status, HR values may often prove to be misleading as indicative and sensitive biomarkers of normal physiology, masking underlying hypotensive states or cardiac contractile downregulation. As discussed earlier, MAP is a more appropriate biomarker, while the combination of both HR and MAP is even more useful as a potential indicator of myocardial oxygen extraction.

Catheters provide the means to monitor intraventricular pressures or monitor hemodynamics during the cardiac cycle; however, there are technical challenges (inherent limitations, insertion and placement, surgical complexities, MR field inhomogeneity artifacts), and their placement imposes physiological risks and prolongation of surgical and anesthetic administration. Unfortunately, there are no other commercially available sensors at present that could easily monitor MAP during imaging studies.

### Molecular and Multinuclear Imaging

For most imaging protocols for molecular and multinuclear MRI, use of inhalational anesthetics has become practically highly desirable. For example, from a scientific standpoint, ISO administration for *in vivo* studies of sodium (^23^Na), phosphorus (^31^P), or potassium (^39^K) is appropriate. However, there could be potential interference of the ISO resonance(s) with ^19^F fluorinated compounds in recent advancements of molecular imaging with exogenously administered fluorinated particles (108, 109). In these cases, alternative strategies of minimizing or eliminating the interference can be successfully implemented through selective suppression or excitation strategies, or redesign/resynthesis of molecular probes to achieve spectral shifts that are far from the deleterious contamination of anesthesia.

## Conclusion

This review has overviewed the emergence and historical evolution of major scientific advancements of anesthetics, including the receptor-mediated effects on brain, heart, and vasculature, and has discussed possible signaling mechanisms triggered by inhalational anesthetics. At the organ level, perfusion, metabolism, and global cardiac functional effects have been considered, with a focus on the paradigm of MR-based rodent imaging where prolonged protocols of study are used, aiming to the establishment of optimal, regional, and global indices of cardiac performance.

## Declaration

Experimental studies and data presented in this review were carried out and collected in accordance with the recommendations of Veterinary Services of the Ministry of Agriculture of the Government of Cyprus. The protocols were approved by the Veterinary Services of the Ministry of Agriculture of the Government of Cyprus in accordance with national rules set by the Ministry, European Animal Research directives, and international guidelines for animal research (NRC 1996). All experiments conformed to the European Convention for the Protection of Vertebrate Animals Used for Experimental and Other Scientific Purposes.

## Author Contributions

CC and KM agree to be accountable for the content of the work. Specific contributions for this work are as follows: CC and KM: conception and design, analysis and interpretation of data, revising the article critically for important intellectual content, final approval of the version to be published, and agreement to be accountable for all aspects of the work in ensuring that questions related to the accuracy or integrity of any part of the work are appropriately investigated and resolved. CC: acquisition of data and drafting the article.

## Conflict of Interest Statement

The authors declare that the research was conducted in the absence of any commercial or financial relationships that could be construed as a potential conflict of interest. The reviewer YB and handling Editor declared their shared affiliation, and the handling Editor states that the process nevertheless met the standards of a fair and objective review.
